# Short CAG repeat variation as a regulatory factor in health and disease

**DOI:** 10.3389/fgene.2026.1885864

**Published:** 2026-06-19

**Authors:** Jacob R. Manjarrez

**Affiliations:** Oklahoma State University Center for Health Sciences, Biochemistry and Microbiology, Tulsa, OK, United States

**Keywords:** androgen receptor, CAG repeats, cytosine-adenine-guanine, Huntington’s disease, polyQ

## Abstract

Short cytosine-adenine-guanine (CAG) trinucleotide repeats, which encode polyglutamine (polyQ) tracts, are prevalent features of genes enriched in transcriptional and regulatory functions, including the androgen receptor (AR) and huntingtin (HTT). While expanded CAG repeats are well established in neurodegenerative disease pathogenesis, the functional significance of short, non-pathogenic repeat lengths remains underappreciated. This review connects evidence demonstrating that short CAG/polyQ tracts act as dynamic modulators of protein conformation, transcriptional activity, and protein-protein interactions. Variation within physiological repeat ranges influences receptor sensitivity, cellular signaling, and phenotypic diversity. The AR serves as a central model, where shorter repeat lengths enhance transactivation and androgen responsiveness and are associated with increased prostate cancer risk, while longer non-expanded repeats are linked to reduced receptor activity and modest reproductive and metabolic effects. Mechanistically, repeat length and sequence composition jointly influence repeat stability, RNA structure, and downstream regulatory processes. Beyond AR, short CAG variation contributes to neuropsychiatric phenotypes and broader regulatory networks. Collectively, short CAG repeats function as quantitative regulators of gene activity, shaping disease susceptibility, physiological variation, and evolutionary adaptation.

## 1 Introduction

Short cytosine–adenine–guanine (CAG) nucleotide repeats, which encode polyglutamine (polyQ) tracts, are a widespread feature enriched in genes encoding transcription factors and regulatory proteins, including the androgen receptor (AR) and huntingtin (HTT) genes. Within physiological ranges, polyQ tracts typically consist of approximately 5–20 glutamine residues. Evidence indicates that repeat lengths below gene-specific pathogenic thresholds are functionally distinct from the longer expansions (generally >40 repeats) that cause repeat-associated neurodegenerative disorders (Ding et al., 2004; Beilin et al., 2000). Rather than being intrinsically pathogenic, short polyQ tracts serve as modulatory elements that influence protein conformation, protein–protein interactions, transcriptional activity, and cellular signaling (Guo et al., 2017). Variation in tract length contributes to differences in receptor sensitivity, localization, and regulatory capacity, highlighting their roles in normal physiology and adaptation.

The AR represents the most extensively studied example of the biological and clinical relevance of short CAG repeats. Within the common range of 11–36 repeats, shorter alleles (<22–23) are associated with increased receptor transactivation and enhanced androgen sensitivity. Epidemiological and case–control studies across diverse populations consistently link shorter AR CAG repeats to increased prostate cancer risk (Murmann et al., 2022; Sun et al., 2023; Cadden et al., 2024; McGurk et al., 2021; Ersoy, 2021; Gudde et al., 2017; Balic et al., 2002; Nelson and Witte, 2002; Sun and Lee, 2013; Qin et al., 2017; Weng et al., 2017; Hsing et al., 2002; Stanford et al., 1997).

Beyond prostate cancer, AR CAG repeat length influences broader physiological and immunological processes. Shorter polyQ tracts may alter immune and inflammatory responses by modulating the transcription of androgen-responsive genes involved in cytokine signaling and immune cell function (Guo et al., 2017; Krishnaswamy et al., 2006; Giovann et al., 1997; Gómez et al., 2016; Hakimi et al., 1997). These findings indicate systemic effects extending beyond reproductive tissues.

Conversely, longer but non-expanded AR CAG repeats are associated with reduced androgen sensitivity and show a modest link to impaired spermatogenesis and male infertility (Li et al., 2011). These observations highlight bidirectional functional consequences of repeat length variation. Repeat stability is not determined solely by repeat number; sequence interruptions within CAG tracts can stabilize these regions and reduce expansion risk (Buchanan et al., 2004).

The functional relevance of short CAG repeats extends beyond androgen signaling. PolyQ length variation has been implicated in transcriptional regulation, neurodegenerative mechanisms, and behavioral and personality traits (Wang et al., 2004; Shi et al., 2011). Together, these findings indicate that short CAG repeats are dynamic regulatory elements rather than neutral polymorphisms. This review synthesizes current evidence supporting their roles in disease susceptibility, physiological variation, and human adaptation.

## 2 Literature search and study selection

A comprehensive literature search was conducted using the Consensus platform, along with manual searches, integrating sources such as Semantic Scholar and PubMed. Two complementary strategies focused on the search terms short polyQ tracts, short polyQ repeats, and short CAG repeats to capture molecular, structural, evolutionary, and clinical studies, including associations with prostate cancer, infertility, and neurodegenerative diseases, using unfiltered searches that contain limited reviews. Relevant studies were selected based on citation impact, recency, and journal quality, following standard identification, screening, eligibility, and inclusion procedures (Supplementary Figure Sl).

### 2.1 Short CAG repeats: nucleotide-level effects and androgen receptor–associated phenotypes

#### 2.1.1 Molecular mechanisms and repeat stability

Short, uninterrupted CAG repeats in the AR gene increase transcriptional activity, resulting in enhanced androgen responsiveness in target tissues (Ding et al., 2004; Beilin et al., 2000). At the nucleic acid level, uninterrupted CAG tracts readily form stable hairpin structures. In polyglutamine disorders such as HD, these secondary structures can be processed into toxic small RNAs through RNA interference pathways, with longer uninterrupted stretches increasing toxicity and accelerating disease onset (Guo et al., 2017; Murmann et al., 2022; Sun et al., 2023).

Sequence interruptions within CAG repeats, such as the inclusion of single CAA codons, substantially alter repeat dynamics by slowing branch migration, stabilizing alternative secondary structures, and reducing replication slippage and pathological expansion. These interruptions, therefore, act as protective modifiers of repeat instability and disease risk (Cadden et al., 2024). This modifying effect is well illustrated in the *ATXN2* gene, where disease association depends not only on repeat length but also on repeat composition. Pure CAG expansions are classically linked to spinocerebellar ataxia type 2 (SCA2). In contrast, interruption of CAG repeats by CAA codons is more commonly associated with amyotrophic lateral sclerosis (ALS) or Parkinson’s disease (McGurk et al., 2021).

Despite these modifying effects, many polyglutamine disorders, including those involving the AR gene, HD, ALS, spinocerebellar ataxias (types 1, 2, 3, 6, 7, and 17), dentatorubral-pallidoluysian atrophy, and spinal and bulbar muscular atrophy, remain strongly correlated with the absolute number of CAG triplet repeats (Ersoy, 2021). Accordingly, repeat length remains a dominant determinant of disease risk and severity, even in the presence of sequence interruptions.

Beyond coding effects, CAG repeats located near or within polyadenylation sites have been shown to influence RNA processing, and alternative splicing events remove expanded CAG tracts as introns (Gudde et al., 2017). These repeats also appear to upregulate myotonic dystrophy type 1 antisense transcripts in patient samples, although their low overall abundance complicates interpretation of their regulatory impact. Collectively, these findings suggest that expanded CAG regions may exert additional molecular effects on transcript stability, splicing, and polyadenylation when positioned within or near 3′regulatory regions (Gudde et al., 2017).

#### 2.1.2 Prostate cancer risk and tumor characteristics

Meta-analyses consistently demonstrate that men with shorter AR CAG repeats, commonly defined as fewer than 22 or 23 repeats, have a modest but statistically significant increase in prostate cancer risk compared with those carrying longer alleles (Ding et al., 2004; Balic et al., 2002; Nelson and Witte, 2002; Sun and Lee, 2013; Qin et al., 2017; Weng et al., 2017; Hsing et al., 2002; Stanford et al., 1997; Krishnaswamy et al., 2006). This association has been observed across multiple ethnic populations, including Caucasian, Asian, Hispanic, Chinese, Mexican, and South Indian cohorts, and is particularly pronounced for high-grade or advanced-stage tumors (Balic et al., 2002; Giovann et al., 1997; Gómez et al., 2016; Hakimi et al., 1997).

Mechanistically, shorter AR CAG repeats enhance androgen signaling and are associated with increased Wnt/β-catenin-driven tumorigenesis, providing a functional basis for the observed epidemiological associations (Li et al., 2011; Buchanan et al., 2004; Wang et al., 2004; Shi et al., 2011; He et al., 2020; Roggero et al., 2022; Platz et al., 2005). Nonetheless, some studies report no significant association in specific populations or when alternative repeat-length cutoffs are applied, highlighting heterogeneity across cohorts (Bratt et al., 1999; Chen et al., 2016; Price et al., 2010).

#### 2.1.3 Male infertility, reproductive, and cardiac health

Longer AR CAG tracts have been modestly associated with idiopathic male infertility, with infertile men exhibiting slightly longer mean (above 40) repeats than fertile controls (Davis-Dao et al., 2007; Tse et al., 2003). However, several studies report no significant differences in fertility outcomes within the normal CAG repeat range (approximately 14–33 repeats), suggesting that repeat length alone is not a dominant determinant of male infertility (Meyts et al., 2002; Sharestani et al., 2021). In otherwise healthy men, shorter AR alleles have been correlated with higher sperm output, although these associations do not independently account for infertility risk (Giovannucci et al., 1999; Von et al., 2001). Supporting this, a Russian cohort study reported that shorter CAG repeats were associated with (Mitsumori et al., 1999) improved sperm parameters, including total count, concentration, progressive motility, and morphology, compared with longer CAG alleles (Osadchuk et al., 2022). In contrast, a separate study found no association between AR CAG length and testosterone levels in men with a median repeat length of 20, highlighting population-specific variability and the influence of additional modifiers, which can also increase the risk for testicular cancer in those with ≥25 repeats (Wang et al., 2021; Grassetti et al., 2015).

The study by Wang et al. (2021) further extended these observations to cardiovascular outcomes, reporting no association between AR CAG length and myocardial infarction (MI) prognosis. Conversely, a Chinese cohort study identified a protective effect associated with AR CAG repeat lengths below 26, with notable sex-specific differences that underscored the need for larger female cohorts to clarify these differences (Zhen et al., 2023). These contrasting results suggest that the cardiovascular impact of AR CAG length may depend on population genetics, sex, and interacting metabolic factors.

AR CAG repeat length has also been implicated in metabolic regulation and insulin sensitivity. Shorter AR tracts (below 23 repeats) have been associated with a modest detrimental effect on insulin resistance, whereas longer CAG repeats appear to confer relative metabolic protection (Möhlig et al., 2011). In a Chinese diabetic cohort, AR CAG length itself was not correlated with cardiovascular or chronic kidney disease risk; however, elevated testosterone levels in individuals with shorter CAG repeats were associated with increased mortality, indicating a complex interaction between androgen signaling and metabolic disease outcomes (Wong et al., 2019). Further supporting this complexity, components of metabolic syndrome, including fasting glucose, C-peptide, and glycosylated hemoglobin (HbA1c), were inversely associated among individuals carrying ≤21 CAG repeats, suggesting repeat-length-dependent modulation of metabolic risk markers rather than uniform disease susceptibility (Skjærpe et al., 2010).

#### 2.1.4 Other hormone-related disease associations

Short AR CAG repeats have been associated with increased risk or severity of several hormone-dependent conditions. In benign prostatic hyperplasia, shorter repeats correlate with larger adenoma size and more severe lower urinary tract symptoms (Krishnaswamy et al., 2006; Möhlig et al., 2011; Wong et al., 2019). With respect to metabolic health, shorter CAG and GGN repeats in the AR gene have been associated with lower adiposity. Specifically, individuals with shorter CAG repeats exhibit reduced total body fat accumulation, lower resting metabolic rate, and decreased lean mass compared with those carrying longer CAG alleles (Ponce‐González et al., 2016).

In breast cancer, fewer AR CAG repeats are associated with more aggressive disease phenotypes, consistent with enhanced AR transcriptional activity (Yu et al., 2000). Short AR alleles have also been linked to increased susceptibility to polycystic ovary syndrome (PCOS), potentially through heightened AR signaling and resultant hyperandrogenism (Schüring et al., 2012; Rajender et al., 2013; Wang, 2012). More recently, shorter AR CAG tracts have been associated with higher antral follicle counts in women with PCOS, increasing the likelihood of polycystic ovarian morphology (Yan et al., 2023). The incidence and prevalence of PCOS have been studied across regions, including the Middle East, the Mediterranean, India, South Asia, Norway, and the United States, and across multiple ethnic populations, including Caucasian, Asian, Hispanic, and Black cohorts, with findings that demonstrate regional and ethnic differences (Vanhise et al., 2023). Another complex issue in women’s health is understanding the pathology of recurrent spontaneous abortion (RSA). Studies examining the androgen receptor (AR) have suggested an increased risk associated with shorter CAG repeat lengths (Ciarloni et al., 2025; Sui et al., 2022). However, current evidence remains inconsistent, with some findings linking both shorter and longer CAG repeats to an elevated risk of RSA (Ciarloni et al., 2025). Because these studies have been conducted across diverse populations, the varying results may reflect underlying ethnic differences in genetic expression (Ciarloni et al., 2025). More targeted and population-specific research is needed to clarify these associations and improve our understanding of RSA.

Beyond reproductive disorders, AR CAG length has been implicated in immune-mediated and sexual health conditions. In women with rheumatoid arthritis, shorter AR CAG repeats are associated with earlier disease onset and increased disease severity (Dziedziejko et al., 2012). Shorter AR CAG lengths have also been linked to female sexual dysfunction; a cross-sectional study reported that women with shorter repeats experienced greater difficulty achieving orgasm (Wåhlin-Jacobsen et al., 2018). In men, the AR CAG, but not the GGN polymorphism, has been associated with sexual dysfunction (Ciarloni et al., 2025).

Conversely, longer AR CAG repeats, while associated with reduced AR activity, have been linked to increased susceptibility to severe COVID-19 outcomes. This association has been proposed to reflect impaired androgen-mediated anti-inflammatory and immunomodulatory signaling in individuals with reduced AR transactivation capacity (Baldassarri et al., 2021). Another reported benefit of shorter CAG repeats is a reduced risk of bone fractures (Ciarloni et al., 2025; Yanovich et al., 2011). However, findings regarding the relationship between shorter CAG repeats, the GGN polymorphism, and decreased bone density remain inconsistent (Yanovich et al., 2011).

#### 2.1.5 Short CAG disease associations beyond the AR

Short CAG repeat lengths are associated with neuropsychiatric phenotypes beyond AR–related disorders. Within the *HTT* gene, shorter CAG repeats have been linked to an increased risk of depression, as demonstrated in a large population-based cohort study (Gardiner et al., 2017a; Moschini et al., 2022). More recently, shorter *HTT* CAG lengths are connected with anxiety disorders, with a similar trend observed for major depressive disorder among individuals carrying fewer than 21 repeats (Vater et al., 2025). These findings suggest that shorter length of pathogenically relevant CAG variation in *HTT* may influence affective and anxiety-related traits independently of Huntington’s disease onset.

Population studies indicate that these shorter repeat lengths are common. Analysis of the Danish national registry revealed a median *HTT* repeat length of 18 and a mean of 19 ± 3.4 repeats within the general population (Gilling et al., 2017). Comparable repeat lengths were also prevalent in a Chinese family-based study examining Huntington’s disease within families, indicating consistency across ethnically distinct populations (Yu et al., 2014). Together, these data highlight that CAG lengths associated with neuropsychiatric phenotypes fall well within the normal population distribution.

Importantly, genetic modifiers can influence the biological consequences of CAG repeat length. Increased expression of FANCD2-and FANCI-associated nuclease 1 (FAN1) has been shown to exert a protective effect in individuals with extended CAG repeat regions, reducing somatic instability and delaying disease progression (Hong et al., 2024; Lee et al., 2019; Goold et al., 2019). The findings emphasize that repeat length alone does not fully determine pathogenic risk and that DNA repair pathways play a critical modulatory role.

**FIGURE 1:**
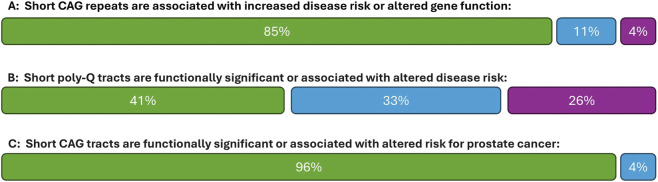
Altered disease or functional risk associated with CAG or Poly-Q tracts based on the papers referenced in this review. Green–Yes, Blue–undecided, and Purple–No, **(A)** n = 55; **(B)** n = 39; **(C)** n = 23.

The influential complexity is further underscored by evidence suggesting that uninterrupted CAG repeat length may not be the sole driver of somatic expansion. Data from the Genetic Modifiers of Huntington’s Disease (GeM-HD) Consortium indicate that repeat instability does not always scale directly with uninterrupted CAG length, raising the question of whether polyglutamine tract length or nucleotide-level repeat structure more accurately predicts disease progression and pathogenesis (Lee et al., 2019). This distinction has important implications for understanding genotype-phenotype relationships across CAG repeat disorders (Figure 1).

Notably, the association between shorter CAG repeats and reduced neuropsychiatric risk is not uniform across genes. Shorter CAG lengths in the repeat-containing genes *ATXN7* and *TBP* have been linked to a decreased lifetime risk of depression, contrasting with observations in *HTT* and suggesting gene-specific effects of CAG repeat variation (Gardiner et al., 2017a; Vater et al., 2025; Gardiner et al., 2017b). Collectively, these findings indicate that the phenotypic consequences of short CAG repeats depend on genomic context, regulatory environment, and interaction with modifier genes, rather than reflecting a universal protective or deleterious effect.

### 2.2 Short poly-Q tracts: protein-level structure, function, and evolutionary effects

#### 2.2.1 Functional roles in transcription and protein interactions

Poly-Q tracts are commonly embedded in transcription factors and regulatory proteins, where they modulate transcriptional activity, protein–protein interactions, and regulatory flexibility (Atanesyan et al., 2012; Escobedo et al., 2018; Cooper and Fassler, 2022). The tracts can stabilize coiled-coil domains or function as flexible interaction modules that fine-tune transcriptional complexes (Cooper and Fassler, 2022; Schaefer et al., 2012).

In yeast Med15 and plant ANGUSTIFOLIA proteins, variation in short poly-Q tract length alters interaction strength with partner transcription factors and affects subcellular localization (Cooper and Fassler, 2022; Bryan et al., 2018). In HTT, modest variation within the physiological poly-Q range correlates with enhanced neural development features, including synaptogenesis and increased brain complexity (Iennaco et al., 2022; Świtońska-Kurkowska et al., 2021).

#### 2.2.2 Structural properties and aggregation behavior

Biophysical studies indicate that polyQ peptides, typically containing fewer than 20 glutamine residues, preferentially adopt random coil or α-helical conformations. These structures are stabilized by unconventional hydrogen bonding between glutamine side chains and backbone carbonyl groups, rather than the interstrand hydrogen bonding characteristic of β-sheet assemblies (Escobedo et al., 2018; Escobedo et al., 2020; Nanajkar et al., 2024). Helicity and conformational stability increase with tract length but remain structurally distinct from the β-sheet–rich aggregates that represent pathogenic polyQ expansions (Chen et al., 2016; Escobedo et al., 2018; Bravo-Arredondo et al., 2018; Barbosa Pereira et al., 2023; Moldovean-Cioroianu, 2024).

#### 2.2.3 Disease associations beyond the AR

Functionally, short polyQ tracts can enhance protein–protein interactions without inducing aggregation. For example, the transcriptional co-regulator ZMIZ1 preferentially interacts with short polyQ regions, leading to upregulation of AR activity (Li et al., 2011). Consistent with this functional compatibility, short polyQ tracts aggregate slowly and do not readily form amyloid-like fibrils. Only when a critical length threshold is exceeded do polyQ sequences undergo rapid conformational transitions that promote fibril formation and pathological aggregation (Jayaraman et al., 2012; Dromb et al., 2018; Scarafone et al., 2012; Urbanek et al., 2020).

Collectively, these findings help explain why short polyQ tracts are generally compatible with normal protein structure and function, while remaining sensitive to modest length variation that can subtly influence conformational dynamics, interaction specificity, and downstream biological activity.

#### 2.2.4 Evolutionary significance and adaptive potential

At an evolutionary level, polyQ repeat length exhibits differential variability depending on tract size and conservation. Shorter polyQ regions display a broader range of length variation, whereas longer polyQ tracts are more prone to contraction events, including deletions. Notably, polyQ regions that are more highly conserved across the animal kingdom tend to exhibit reduced length variability, suggesting functional constraints on repeat expansion or contraction in these contexts (Mier et al., 2024).

Comparative genomics indicates that short CAG-encoded poly-Q tracts are under purifying selection in key regulatory proteins such as HTT, although small length variations can drive adaptive changes in neural function across species (Iennaco et al., 2022; Mier and Andrade-Navarro, 2018; Mier et al., 2021). Codon usage analyses further reveal that CAG triplets are more prevalent in short glutamine homo-repeats than in longer tracts, suggesting a mechanism for evolutionary expansion while maintaining functional stability (Mier and Andrade-Navarro, 2018).

## 3 Discussion

Collectively, the evidence supports a model in which short CAG repeats and their encoded polyQ tracts function as adaptable regulatory elements that modulate protein activity, interaction networks, and disease susceptibility across biological systems (Table 1). These tracts are enriched in transcription factors and regulatory proteins, where they enhance protein–protein interactions and transcriptional output without inducing aggregation characteristic of pathogenic expansions (Goold et al., 2019; Gardiner et al., 2017b; Bryan et al., 2018). Subtle variation in polyQ length therefore fine-tunes regulatory capacity while remaining compatible with normal cellular function.

**TABLE 1:** Selective key categories and key findings synthesized from the reviewed literature.

Category	Key findings	References
Prostate cancer risk	• Shorter AR CAG repeats (≤21) significantly increase prostate cancer risk • Association consistent across diverse populations (Swedish, Hispanic, Indian, Chinese, Mexican) • Short CAG repeats enhance AR transcriptional activity specifically in prostate cells • Coactivator recruitment enhanced by short polyQ tracts • Multiple repeat polymorphisms (CAG, GGN) contribute to risk • Short repeats linked to disease progression and therapy response	Beilin et al. (2000), Balic et al. (2002), Sun and Lee (2013), Hsing et al. (2002), Gómez et al. (2016), Li et al. (2011), Buchanan et al. (2004), He et al. (2020), Bratt et al. (1999), Chen et al. (2016), Price et al. (2010), Schüring et al. (2012), Andersson et al. (2006), Zeegers et al. (2004)
Molecular mechanisms and structural biology	• PolyQ tracts show length-dependent conformational plasticity and structural flexibility • PolyQ length influences protein folding, aggregation, and transcriptional regulation • PolyQ tracts mediate protein-protein interactions and coactivator recruitment • RNA secondary structures formed by CAG repeats contribute to toxicity and regulation • Interruptions in CAG repeats reduce toxicity and aggregation propensity	Murmann et al. (2022), Cadden et al. (2024), Li et al. (2011), Shi et al. (2011), He et al. (2020), Escobedo et al. (2018), Cooper and Fassler (2022), Escobedo et al. (2020), Escobedo et al. (2020), Barbosa Pereira et al. (2023), Jayaraman et al. (2012), Dromb et al. (2018), Urbanek et al. (2020)
Neurodegeneration and Huntington’s disease	• CAG repeat length and purity influence Huntington’s disease onset and progression • PolyQ aggregation proceeds through structured oligomeric intermediates • Genetic modifiers can stabilize expanded repeats and modify the disease course • Short CAG is involved in increased depression risk • PolyQ diseases share neurodevelopmental and neurodegenerative features	Gardiner et al. (2017a), Hong et al. (2024), Lee et al. (2019), Gardiner et al. (2017b), Iennaco et al. (2022), Świtońska-Kurkowska et al. (2021), Nanajkar et al. (2024), Dromb et al. (2018), Mohanty et al. (2025)
Clinical and metabolic effects	• CAG repeat length influences insulin sensitivity, fat accumulation, metabolic syndrome, and cardiovascular risk • Effects are hormone-dependent and sex-specific • CAG polymorphisms affect reproductive health including male infertility and female endocrine diseases (PCOS) • Short repeats associated with altered sexual function and fertility	Davis-Dao et al. (2007), Tse et al. (2003), Sharestani et al. (2021), Wang et al. (2021), Möhlig et al. (2011), Skjærpe et al. (2010), Schüring et al. (2012), Rajender et al. (2013), Yan et al. (2023), Wåhlin-Jacobsen et al. (2018), Deng et al. (2017)
Bidirectional health effects	• Short CAG repeats increase prostate cancer risk but protect against severe COVID-19 in males • Short repeats also enhance transcriptional activity but reduce fertility • Effects depend on cell type, tissue context, and hormonal environment	Beilin et al. (2000), Nelson and Witte (2002), Baldassarri et al. (2021), Andersson et al. (2006), Undurraga et al. (2012), Rice et al. (2015)
Evolutionary and functional role of PolyQ	• PolyQ tracts are conserved transcriptional modulators across species • Length-dependent regulation of transcriptional activity • PolyQ repeats serve as protein interaction hubs and network nodes • Evolutionary optimization balances function and aggregation risk	Atanesyan et al., 2012, Schaefer et al. (2012), Mier and Andrade-Navarro (2018), Mier et al. (2021), Ashkenazi et al. (2017)

Structural studies provide a mechanistic basis for these effects. Short polyQ segments adopt non-β-sheet conformations distinct from amyloid aggregates observed at pathogenic lengths (Lee et al., 2019; Bryan et al., 2018). Only beyond a threshold do polyQ tracts shift toward aggregation-prone conformations associated with neurodegenerative disease (Sharestani et al., 2021; Świtońska-Kurkowska et al., 2021). Even within non-pathogenic ranges, modest variation in repeat length can influence protein stability and interaction dynamics.

The AR represents the most extensively studied example of short CAG/polyQ variation. Evidence demonstrates that shorter uninterrupted CAG tracts increase AR transcriptional activity, amplifying androgen signaling (Guo et al., 2017; Krishnaswamy et al., 2006; Giovann et al., 1997; Gómez et al., 2016). Consistent with this mechanism, meta-analyses across diverse populations show that men with shorter AR CAG repeats (<22–23) have a modest but statistically significant increase in prostate cancer risk (Murmann et al., 2022; Sun et al., 2023; Gudde et al., 2017; Balic et al., 2002; Nelson and Witte, 2002; Sun and Lee, 2013; Urbanek et al., 2020). Although effect sizes are small, the association is consistent across studies (Qin et al., 2017; Mier et al., 2024). Heterogeneity across cohorts indicates that AR CAG repeat length functions as a modifying risk factor, influenced by genetic, hormonal, and environmental factors (Sharestani et al., 2021; Von et al., 2001).

Beyond prostate cancer, AR CAG repeat variation influences hormone-dependent conditions. Shorter repeats are associated with increased severity in benign prostatic hyperplasia, aggressive breast cancer phenotypes, and polycystic ovary syndrome (Schüring et al., 2012; Wang, 2012; Yan et al., 2023), while longer repeats have been linked to increased susceptibility to severe COVID-19 outcomes (Baldassarri et al., 2021). These findings support a bidirectional and context-dependent model of repeat-length effects.

In reproductive physiology, longer AR CAG repeats show a weak association with idiopathic male infertility, reinforcing the view that repeat length primarily modulates physiological traits rather than acting as a primary disease driver (Li et al., 2011; Osadchuk et al., 2022; Wang et al., 2021).

Evolutionary analyses further demonstrate that short CAG/polyQ tracts are subject to strong selective constraints. In conserved proteins such as HTT, small length variations are maintained across species and contribute to functional diversity while avoiding aggregation (Cooper and Fassler, 2022; Dromb et al., 2018; Scarafone et al., 2012). At the nucleotide level, uninterrupted CAG tracts influence RNA structure and processing, while sequence interruptions stabilize repeats and reduce expansion risk (Buchanan et al., 2004; Roggero et al., 2022; Platz et al., 2005). Both expansion and contraction outside an optimal range can disrupt function, highlighting a narrow evolutionary tolerance window (Mier and Andrade-Navarro, 2018; Mier et al., 2021).

Overall, short CAG repeats and polyQ tracts act as quantitative regulators of protein function (Figure 1), influencing disease susceptibility, physiological variation, and adaptation without inducing overt protein misfolding. Their dual role—functional at physiological lengths yet destabilizing when perturbed—helps explain both their evolutionary conservation and association with disease.

Despite increasing recognition, most studies focus on long repeat expansions, often overlooking shorter repeats within datasets. More targeted analyses of short CAG/polyQ tracts are needed to clarify their bidirectional roles and uncover additional clinical implications.

## Supplementary material

SUPPLEMENTARY FIGURE S1 Flow diagram of the literature search and study selection process for short poly-Q tracts and short CAG repeats.
