# Supplementary figures and images for: Short CAG repeat variation as a regulatory factor in health and disease
Source: Front Genet. 2026 Jun 19;17:1885864. doi: 10.3389/fgene.2026.1885864 (PMC13327660; doi:10.3389/fgene.2026.1885864)

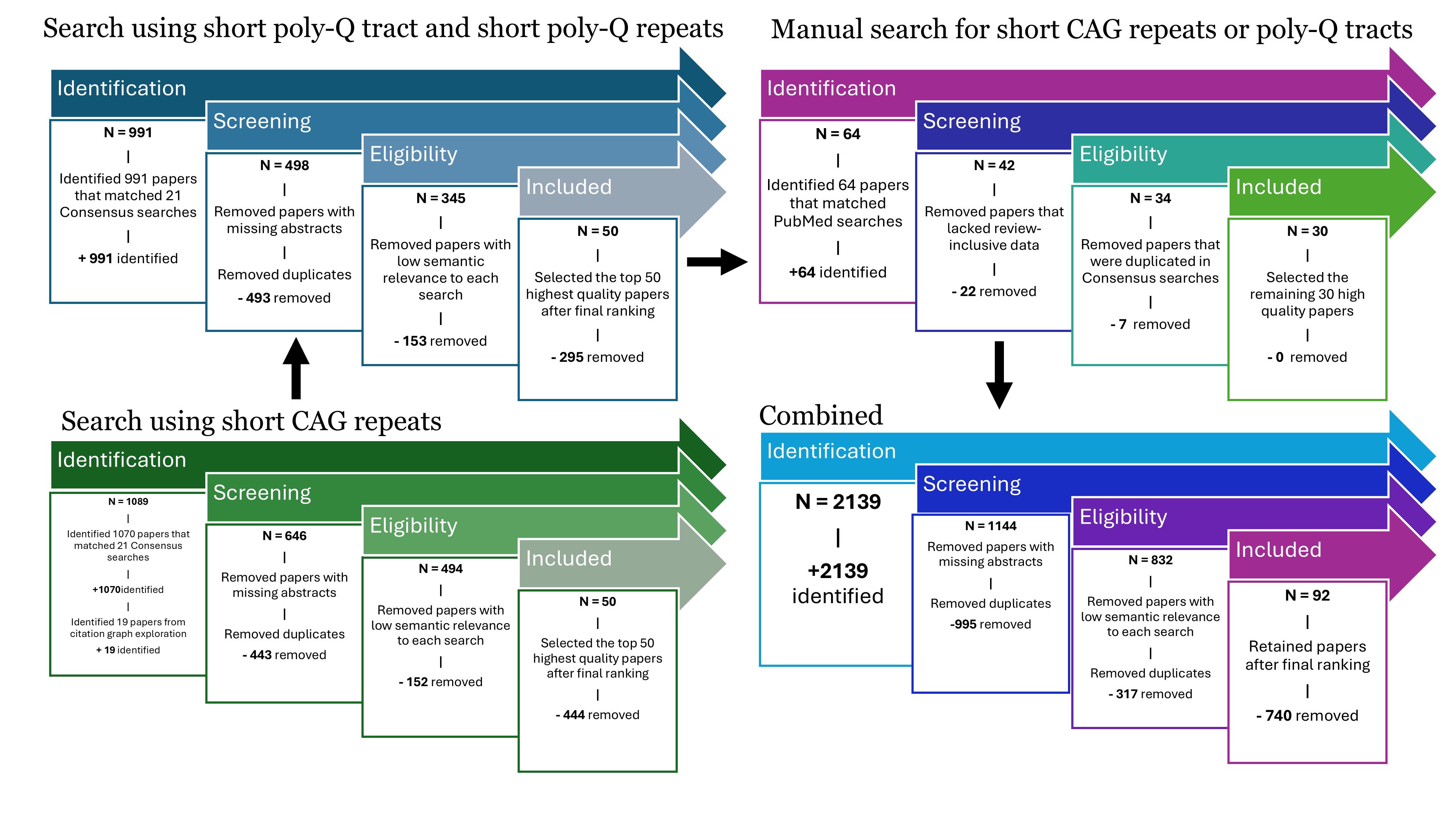

Supplement: Supplementary file 1 [file Image1.jpeg]
